# Indirubin inhibits Wnt/β-catenin signal pathway via promoter demethylation of WIF-1

**DOI:** 10.1186/s12906-020-03045-9

**Published:** 2020-08-14

**Authors:** Shou Gang Liu, Guang Pu Luo, Yong Bin Qu, Yong Feng Chen

**Affiliations:** Dermatology Hospital, Southern Medical University, 2, lujing Road, Yuexiu District, Guangzhou, Guangdong 510091 People’s Republic of China

**Keywords:** Indirubin, Wif-1, DNMT1, Wnt signal pathway, Psoriasis

## Abstract

**Background:**

Psoriasis is a common inflammatory skin disease. Abnormal proliferation of keratinocytes is one of the psoriatic histopathological features. Indirubin has an essential effect on the proliferation and activation of keratinocytes; however, in psoriasis, the specific mechanism of action of indirubin on keratinocytes is unclear. In the present study, we revealed the effects of indirubin on DNA methyltransferase 1 (DNMT1), wnt inhibitory factor 1 (wif-1), and wnt/β-catenin signal pathway, in the meantime, we explored the effects of indirubin on proliferation, cell cycle and the apoptosis of HaCaT cells.

**Methods:**

The expression of DNMT1, wif-1, Frizzled2, Frizzled5, and β-catenin in HaCaT cells treated with different concentrations of indirubin were detected by Western blotting and quantitative real-time polymerase chain reaction (qRT-PCR). The expression levels of DNMT1 and wif-1 were observed after treated with different concentrations of indirubin by enzyme-linked immunosorbent assay (ELISA). The wif-1 promoter methylation status was detected by DNA methylation-specific PCR (MSP). The transcriptional activities of wif-1 and β-catenin were discovered by a luciferase reporter gene system. Cell viability was determined by Cell Counting Kit-8 (CCK8) method. The cell cycle was detected by flow cytometry. The apoptotic cells were surveyed by the apoptosis kit. The expression of Inolucrin, Loricrin, Filaggrin, Keratin 17, and transcriptional activation of transglutaminase 1(TGase1) were detected by Western blotting.

**Results:**

Indirubin inhibited the expression of DNMT1 and the methylation of the wif-1 promoter. In the wnt signal pathway, indirubin restored the protein expression of wif-1 and inhibited expression of Frizzled2, Frizzled5, and β-catenin. Besides, indirubin inhibited the proliferation of HaCaT cells, induced apoptosis, and arrest cell cycle. We also reported that indirubin could down-regulate the expression of Involucrin, TGase 1, and keratin 17, but the expression of Filaggrin and Loricrin had no significant effect.

**Conclusion:**

Our research showed that indirubin promoted the demethylation of wif-1 and suppressed the wnt/β-catenin signal pathway, thereby exerted an anti-proliferative effect. This study reveals the anti-proliferation mechanism of indirubin, which may provide an effective option for the treatment of proliferative diseases.

## Background

Psoriasis is a chronic inflammatory skin disease, which is characterized as erythema or plaques with silvery-white scales, abnormal keratinocyte proliferation, dermal angiogenesis, and immune cell infiltration [1]. It is usually accompanied with multifaceted diseases, such as cardiovascular disease, non-alcoholic fatty liver disease, Crohn’s disease, depression, and metabolic syndrome [2].

The efficacy of traditional treatments for psoriasis, including acitretin and methotrexate, has not been satisfactory. Although biological agents based on the molecular immune mechanism, such as adalimumab, infliximab, and secukinumab, are extensively used, and they have been proved to be extremely useful while there is lack of abundantly long-term safety data. Because of concerns with side effects and the safety of long-term use of these drugs, alternative therapies for psoriasis are receiving increasing attention, including treatment with herbal medicines and pharmaceutic preparation of Chinese herbal extracts.

Indirubin, a bis-indole alkaloid used in traditional Chinese medicine, has long been used to treat various inflammatory disease and dermatosis [3], it has a variety of biological functions that have a vital impact on cell proliferation, activation, and migration [4]. Indirubin plays a crucial role in inhibiting the occurrence and development of psoriasis by inhibiting the activity of EGFR (Epidermal growth factor receptor) [5]. Indirubin treatment can disrupt the progression of IMQ-induced psoriasis-like disease in relevant murine models by reducing γδT cell infiltration, inflammatory cytokine expression, and JAK/STAT signal pathway [6]. It is presently used to treat psoriasis and is safe [7].

In recent years, the potential role of the wnt signal pathway in psoriasis has received increasing attention [8]. Signal transduction pathways are activated to control a variety of cellular processes such as proliferation, apoptosis, migration, and polarity by wnt canonical signaling pathway. By the binding of wnt ligands to the secreted frizzled-related proteins (sFRPs) family of proteins and receptors and the low-density lipoprotein (LDL) receptor-related protein 5 and 6 (LRP5/LRP6) co-receptors initiate the Signaling. The wnt signal pathway plays a significant role in the pathogenesis of psoriasis by regulating the proliferation and differentiation of keratinocytes [9].

Wif-1 is an antagonist of the wnt signal pathway. It binds to wnt ligands, thus inhibiting the wnt signal pathway. Wif-1 mRNA expression is reportedly down-regulated by > 10-fold in psoriatic lesions, and wif-1 immunostaining is also reduced [10]. The expression of wif-1 is related to its methylation status. It has been reported the wif-1 expression is downregulated by DNA hypermethylation in nasopharyngeal carcinoma, breast cancer, and lung cancer. However, the methylation status of wif-1 in psoriasis is unknown.

However, the relationship between indirubin and wnt signal pathway in keratinocytes remains unknown. In this study, the biological effects of indirubin on HaCaT cells was investigated, and a possible mechanism was explored.

## Methods

### Chemicals

Human Phospho-β-Catenin (Ser33/37/Thr41) Antibody (1:1000, #9561, CST, Boston, MA,USA), Human anti-wif-1 antibody (1:1000, #5652, CST), Human anti-DNMT1(1:1000, #5032, CST), Frizzled 5 (1:1000, #5266, CST), Frizzled 2 (1:200, sc-74,019, Santa Cruz, CA, USA), Human anti-GAPDH (1:200, sc-47,724, Santa Cruz), anti-mouse IgG HRP conjugate (1:8000, W402B, Promega, Madison, WI, USA), and anti-Rabbit IgG HRP conjugate (1:2000, #7074P2, CST). Indirubin was obtained from Aladdin (#I132661, Shanghai, China) and dissolved in dimethyl sulfoxide (DMSO).

### Cell culture

The human keratinocyte cell line HaCaT was purchased from Cell Lines Services (Order No. 300493) (Eppelheim, Germany). Cells were cultured in Dulbecco’s Modified Eagle's medium (DMEM) (Gibco, Grand Island, NY, USA) supplemented with 10% fetal bovine serum (FBS) (Gibco) and antibiotics.

### ELISA and Western blotting

The protein levels of DNMT1 and wif-1 were monitored using a human DNMT1 ELISA kit (Bio-Swamp, HM10986, Beijing, China) and a human wif-1 ELISA kit (RayBio® Human WIF- ELISA Kit ELH-WIF-1, RayBiotech corporation). For Western blotting assays, after 48 h treatment with indirubin at different concentrations (0, 0.04 μM, 0.2 μM, and 1 μM), total proteins were collected from RIPA lysis buffer containing protease inhibitor (Sigma-Aldrich, St. Louis, MO, USA). Protein concentrations were measured with a BCA protein assay kit (polyacrylamide gel e, Nanjing, Jiangsu, China). For each protein sample, 30 μg of protein lysate was used for Western blotting. These were separated by 6–10% sodium dodecyl sulfate-polyacrylamide gel electrophoresis and were transferred to a polyvinylidene difluoride membrane. Electrochemiluminescence (KeyGEN BioTECH) was used to visualize the protein bands.

### Quantitative real-time PCR

Total RNA from HaCaT cells was extracted using Trizol (Invitrogen, Carlsbad, CA, USA). Then 1 μg RNA was reverse-transcribed to synthesize cDNA as a template for qRT-PCR using related reverse transcription reagents (Promega, Madison, WI, USA). A SYBR Green PCR Kit (Invitrogen, Carlsbad, CA, USA) was used for amplification via qRT-PCR in a Strata gene MX3000P Sequence Detection System. Glyceraldehyde-3-phosphate dehydrogenase *(GAPDH)* levels were used to normalize gene expression levels in each cDNA sample. The primer sequences used are listed in Table 1.

### Methylation-specific PCR (MSP)

MSP analysis of the human WIF- promoter was performed according to previously described methods [11]. Human genomic DNA from HaCaT cells treated with indirubin was obtained using a genomic DNA kit (TIANGEN Biotech, Beijing, China), then subjected to bisulfite modification using a DNA Methylation Kit (Zymo Research, Orange, CA, USA). The modified DNA (1 μg) was amplified using methylated and unmethylated primers for MSP-which were designed by the Winkybio Company (Guangzhou, China)-under the following conditions: 95 °C for 5 min; 38 cycles of 95 °C for 30 s, 62 °C for 30 s, and 72 °C for 30 s, with a final extension for 10 min at 72 °C. PCR products were evaluated by using 1% agarose gels. Primer sequences used for MSP are listed in Table 1.

**Table 1:** Primers Sequences for qRT-PCR and MSP used in this study

Inolucrin	F-TCAATACCCATCAGGAGCAAATG
	R-GAGCTCGACAGGCACCTTCT
TGase1	F-TCTTCAAGAACCCCCTTCCC
	R-TCTGTAACCCAGAGCCCTTCGA
Keratin17	F-GAGATTGCCACCTACCGC
	R-TGCCATCCTGGACCTGTT
Loricrin	F-TCATGATGCTACCCGAGGTTTG
	R-CAGAACTAGATGCAGCCGGAGA
Filaggrin	F-CTCAGCACAAGGAAGACAGG
	R- TTGTGTTCTGGTGGCTTGTC
GAPDH	F-CGGAGTCAACGGATTTGGTCGTAT
	R-AGCCTTCTCCATGGTGGTGAAGAC
pRL-TK-WIF-1	F-CGGAGTCAACGGATTTGGTCGTAT
	R-AGCCTTCTCCATGGTGGTGAAGAC
Methylated primer	F- 5-CGTTTTATTGGGCGTATCGT-3
	R- 5-ACTAACGCGAACGAAATACGA-3
Unmethylated primer	F- 5-GGGTGTTTTATTGGGTGTATTGT-3
	R-5-AAAAAAACTAACACAAACAAAATACAAAC-3

### Cell viability assay

Cell viability was assayed using Cell Counting Kit-8 (CCK8) (Kumamoto, Japan). The cells were plated in 96-well plates at a density of 3000 cells per well and treated with different concentrations (0, 0.04 μM, 0.2 μM, and 1 μM) of indirubin. After incubation for 48 h, cell viability was assessed. Briefly, 100 μl medium per well was incubated with 10 μl CCK8 solution for 2 h at 37 °C. Finally, absorbance was measured at 450 nm. Optical Density (OD) of the three wells in each group were used to represent the cell proliferation.

### Apoptosis assay

Apoptotic cells were evaluated by Annexin V-fluorescein isothiocyanate (FITC)/propidium iodide (PI) apoptosis assay kit staining (BD Biosciences, San Diego, CA, USA). In brief, HaCaT cells were plated in 6-well plates at a density of 3 × 10^5^ cells per well and treated with indirubin for 48 h. Then, collected cells were washed twice with 1× phosphate-buffered saline (PBS, Sigma-Aldrich), resuspended in binding buffer, and stained with Annexin V-FITC and PI. After incubation in the dark at 4 °C for 15 min, the stained cells were analyzed using a Beckman Coulter Flow Cytometer (Beckman Gallios, Fullerton, CA, USA).

### Cell cycle analysis by flow cytometry

Cells (3 × 10^5^) in 6-well plates were treated with various doses of indirubin. Cells were then trypsinized after 48 h, washed with PBS, and fixed with precooled 70% ethanol at − 20 °C for 12 h in the dark. Before the analysis of the cell cycle, cells were washed with cold PBS again and resuspended with 250 μL propidium iodide (PI) solution containing 10 mg/mL RNase (Thermo Scientific, Lafayette, CO, USA) at 4 °C for 1 h. Finally, cell cycle distributions of all harvest cell samples were measured using a Beckman Coulter Flow Cytometer.

### Luciferase reporter gene assay

To test the effect of indirubin and DNMT1 on the wif-1 promoter, we designed primers based on Noemi Reguart et al. [12], which contain the wif-1 promoter region of MSP and USP (from 1512 to + 6). The fragment was cloned into the pRL-TK vector (Promega) and designated as pRL-TK-WIF-1-Luc. HaCaT cells at 70% confluence were transfected with 200 ng pRL-TK-WIF-1- Luc reporter plasmids and together transfected with 300 ng pRL-TK-DNMT1 plasmid using transfection reagent Lipofectamine 2000 (Invitrogen, Carlsbad, CA). Following 8 h of transfected activity, the transfection complex was replaced with an absolute medium. After 24 h, 1 μM of indirubin was added, and HaCaT cells were lysed over 24 h (Table lists specific promoter fragments). To determine the effect of indirubin on the transcriptional activities of wif-1 and wnt/β-catenin, 200 ng TOP / FOP Flash vector (Gifted by Jiaxin Lin, Guangdong Provincial People’s Hospital, China), 300 ng pRL-TK-WIF-1 plasmid, 20 ng the paL-TK (Promega) vector was transfected into HaCaT cells. The transfected method was the same as the description above. After 24 h, 1 μM of indirubin was added, and HaCaT cells were lysed for 24 h. Luciferase activity was measured using a dual-luciferase reporter assay system (Promega). Transfection experiments were performed in triplicate and repeated three times independently.

### Statistical analysis

Each experiment was performed at least three times. Statistical analyses were performed using Statistical Product and Service Solutions (SPSS) version (SPSS Inc., Chicago, IL, USA). Student’s *t*-test was used for comparisons. Data are displayed as the means ±S.D. *P* values less than 0.05 were considered statistically significant.

## Results

### Indirubin recovered the expression of wif-1 in HaCaT cells

**Fig. 1:**
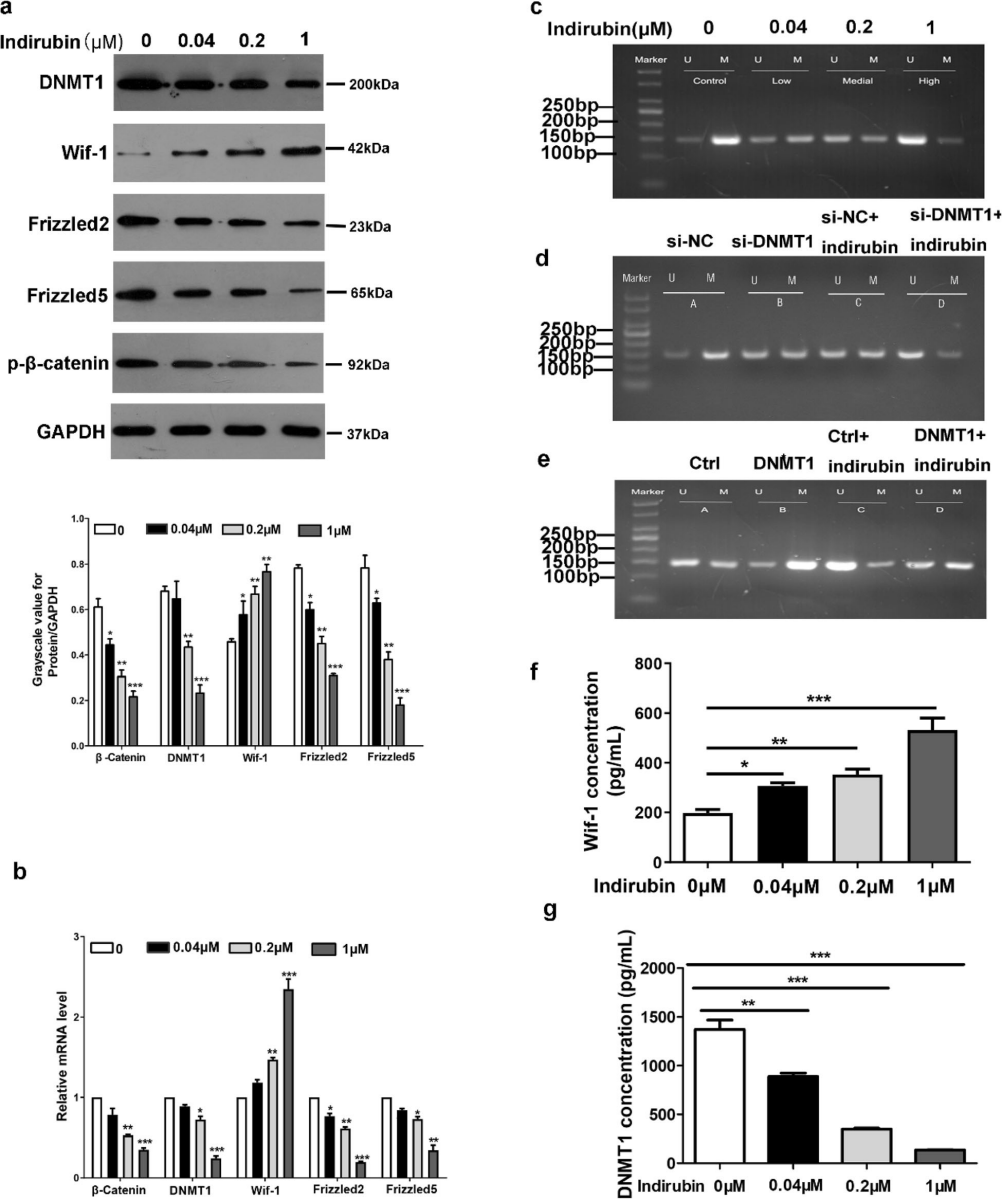
Indirubin inhibits the expression of DNMT1, restores wif-1 expression, and inhibits wnt/β-catenin signal pathway. (**a**): The expression of wif-1 was promoted and the expression of DNMT1, Frizzled2, Frizzled5, and phosphorylation β-catenin was suppressed after treatment with different concentrations (0.04 μM, 0.2 μM, and 1 μM) of indirubin for 48 h compared with the negative control group in HaCaT cells by Western blotting using GAPDH as an internal control. (**b**): The mRNA expression levels of related proteins after treated with different concentrations (0.04 μM, 0.2 μM, and 1 μM) of indirubin. The mRNA expression level of wif-1 was promoted, and the mRNA expression levels of DNMT1, Frizzled2, Frizzled5, and β-catenin were suppressed compared with the negative control group in HaCaT cells by qRT-PCR. (**c**): WIF-1 promoter methylation level decreased after treated with low (0.04 μM), medial (0.2 μM), and high(1 μM) concentrations of indirubin while abnormal methylation was observed in the negative control group. (**d**): The silencing of DNMT1 suppresses wif-1 promoter hypermethylation in HaCaT cells, likewise, wif-1 promoter hypermethylation was suppressed after treated with indirubin(1 μM) together with si-NC, further, wif-1 promoter hypermethylation was significantly suppressed after treated with indirubin(1 μM) together with si-DNMT1, relative to the si-NC group (Fig.1e). (**e**): The overexpression of DNMT1 significantly promoted wif-1 promoter methylation level in HaCaT cells, nevertheless,wif-1 promoter methylation level was suppressed after treated with indirubin(1 μM) together with control,wif-1 promoter methylation level was promoted after treated with indirubin(1 μM) together with DNMT1, relative to the control group. (**f**): The **protein** expression of wif-1 was encouraged after treated with different concentrations (0.04 μM, 0.2 μM, and 1 μM) indirubin compared with the negative control group in HaCaT cells by ELISA. (**g**): The mRNA expression of DNMT1 was suppressed after treated with different concentrations (0.04 μM, 0.2 μM, and 1 μM) of indirubin compared with the negative control group in HaCaT cells by ELISA. (**d**): A: si-NC, B: si-DNMT1, C:si-NC + indirubin, D:si-DNMT1 + indirubin. (**e**): A: Ctrl, B: DNMT1, C: Ctrl+indirubin, D: DNMT1+ indirubin. *P < 0.05, **P < 0.01, ***P < 0.001. U: unmethylated; M: methylated (*N* = 3)

we detected the expression of wif-1 was recovered after the treatment with different concentrations(0.04 μM, 0.2 μM, and 1 μM) of indirubin in a concentration-dependent manner (Fig.1a). Similarly, we observed the mRNA expression of wif-1 was recovered by qRT-PCR (Fig.1b), and protein expression of wif-1 was recovered by ELISA (Fig.1f).

### Indirubin restored wif-1 expression by promoter demethylation in HaCaT cells

We further investigated the effect of indirubin on the methylation status of the wif-1 promoter by MSP. The research showed that wif-1 promoter hypermethylation in HaCaT cells after the treatment with low (0.04 μM), medial (0.2 μM), and high (1 μM) concentrations of indirubin, the wif-1 promoter methylation level was recovered with concentration-dependent demethylation in HaCaT cells (Fig.1c). Otherwise, indirubin upregulated the mRNA expression of wif-1 by qRT-PCR and protein expression of wif-1 by ELISA was observed. These conclusions suggested that indirubin recovered expression of wif-1 by in wif-1 promoter demethylation manner. The results also indicated that the loss expression of wif-1 is associated with wif-1 promoter hypermethylation.

### DNMT1 promotes wif-1 promoter hypermethylation

DNA methylation is an essential epigenetic modification, which is the addition of a methyl group to the base of cytosine by DNA methyltransferase [13]. It is involved in the expression regulation of psoriasis-related genes [14] Methyltransferase DNMT1 as hemi-methylation is considered necessary for maintaining methylation [15]. To further investigate the effect of DNMT1 on the wif-1 promoter methylation, we performed the MSP experiments. We found that the silencing of DNMT1 resulted in wif-1 promoter demethylation (Fig.1d), and overexpression of DNMT1 encouraged wif-1 promoter hypermethylation by MSP (Fig.1e). These conclusions indicate that the presence of DNMT1 promotes wif-1 promoter hypermethylation.

### Indirubin inhibits the expression of DNMT1 and suppresses the expression of wnt/β-catenin signal pathway related proteins

DNMT1 expression is higher in psoriatic peripheral blood mononuclear cells (PBMCs) than in normal controls [16]. We observed in the experiment the expression of DNMT1 was inhibited after treatment with different concentrations(0.04 μM, 0.2 μM, and 1 μM) of indirubin in a concentration-dependent manner by Western blotting (Fig.1a), analogously, the repressed mRNA expression of DNMT1 was observed by qRT-PCR and ELISA, respectively (Fig. 1b and g). Besides, we found that indirubin inhibited expression of phosphorylation β-catenin (Ser33/37/Thr41), Frizzled2, and Frizzled5 by Western blotting (Fig.1a), likewise, indirubin inhibited the mRNA expression of β-catenin, Frizzled2, and Frizzled5 by qRT-PCR. The results suggested that the expression of DNMT1 and related proteins(β-catenin, Frizzled2, and Frizzled5) were suppressed after treatment with different concentrations(0.04 μM, 0.2 μM, and 1 μM) of indirubin in a concentration-dependent manner.

### Indirubin promotes the transcriptional activity of wif-1 and inhibits the transcriptional activity of β-catenin in HaCaT cells

**Fig. 2:**
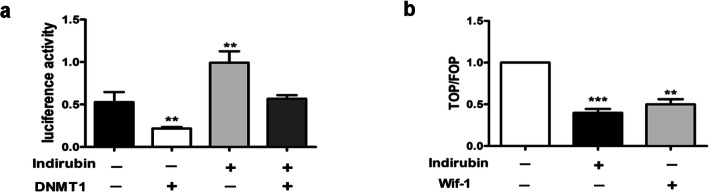
The effect of indirubin on transcriptional activity in wif-1 and β-catenin. (**a**) Luciferase reporter system was used to detect wif-1activity, the results showed concentration raise of pRL-TK-WIF-1-Luc dual luciferase activity after treatment by indirubin(1 μM) compared to the activity in the negative control group, inversely, the concentration reduces of pRL-TK-DNMT-1-Luc dual luciferase activity after exogenously added DNMT1 in HaCaT cells compared to the activity in the negative control group. (**b**) TOP Flash and FOP Flash reporters are widely used to evaluate the β-catenin-dependent wnt/β-catenin signal. Wnt/β-catenin signal activity was inhibited when treatment with indirubin(1 μM), similar results was observed when exogenously added wif-1. **P < 0.01, ***P < 0.001. (*N* = 3)

We explored the transcriptional activity of the wif-1 and Wnt/β-catenin signal pathway respectively. The result showed that indirubin (1 μM) significantly facilitated the luciferase activity of the pRL-TK-WIF-1-Luc plasmid group compared to the negative control group (Fig.2a). The luciferase activity of HaCaT cells in the pRL-TK-DNMT1 plasmid group significantly decreased compared to the activity in the negative control group (Fig.2a). These results indicate that indirubin promotes wif-1 activity, nevertheless, wif-1 activity is suppressed by DNMT1. We employed TOP Flash and FOP Flash reporters, which are widely used to evaluate β-catenin-dependent signaling. The result displayed that activity of Wnt/β-catenin signal was inhibited when treatment with indirubin(1 μM), Similar results was observed when treatment with wif-1(Fig.2b).

### The ability of indirubin inhibits the proliferation, arrests cell cycle, and induces apoptosis of HaCaT cells

**Fig. 3:**
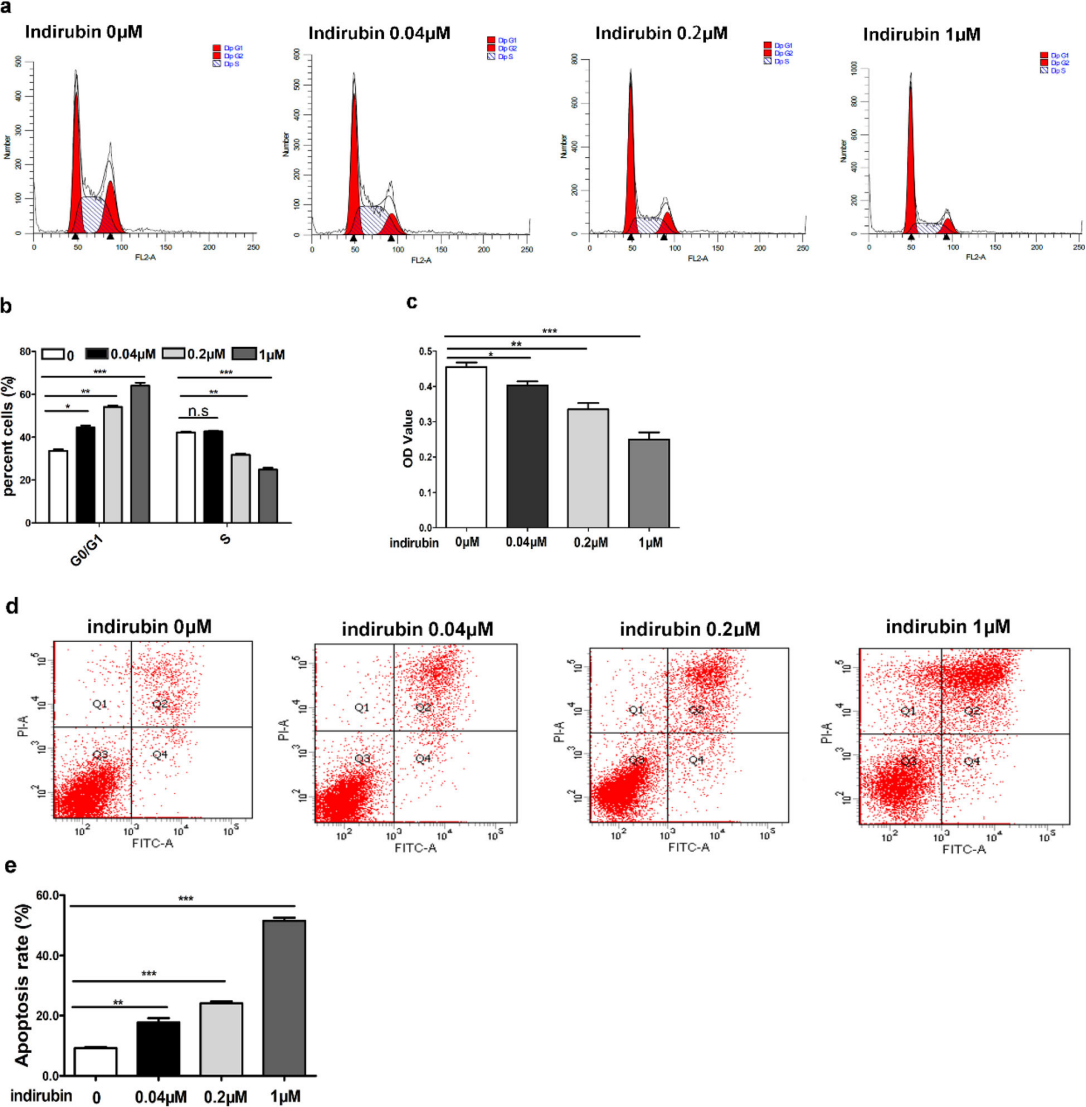
Indirubin inhibits the proliferation and cell cycling of HaCaT cells and induces the apoptosis of HaCaT. (**a** and **b**): The effects of different concentrations (0.04 μM, 0.2 μM, and 1 μM) of indirubin on the cell cycle of HaCaT cells. Indirubin significantly led to the G0 / G1 phase arrest and decreased the proportion of cells in the S phase when the concentration of indirubin was 0.04 μM. (**c**): The proliferation of HaCaT cells was suppressed after treatment with different concentrations (0.04 μM, 0.2 μM, and 1 μM) of indirubin in a concentration-dependent manner. (**d** and **e**): Different concentrations (0.04 μM, 0.2 μM, and 1 μM) of indirubin induced the apoptosis of HaCaT cells in a concentration-dependent manner. *P < 0.05, **P < 0.01, ***P < 0.001. (*N* = 3)

We further explored the effect of indirubin on HaCaT cells. HaCaT cells were exposed to indirubin (concentrated0, 0.04 μM, 0.2 μM, and 1 μM) for 48 h, indirubin at 1 μM increased the percentage of G0/G1 cells from 33.6% in control cells to 64.00% after 48 h of treatment. When the concentration of indirubin was 0.04 μM, the proportion of cells in the S phase began to decrease (Fig.3a and b). The proliferation of HaCaT cells decreased with increasing concentration of indirubin (Fig.3c), and apoptosis was induced (Fig. 3 d and e).

### Indirubin treatment reduces the expression of crucial proteins in HaCaT cells

**Fig. 4:**
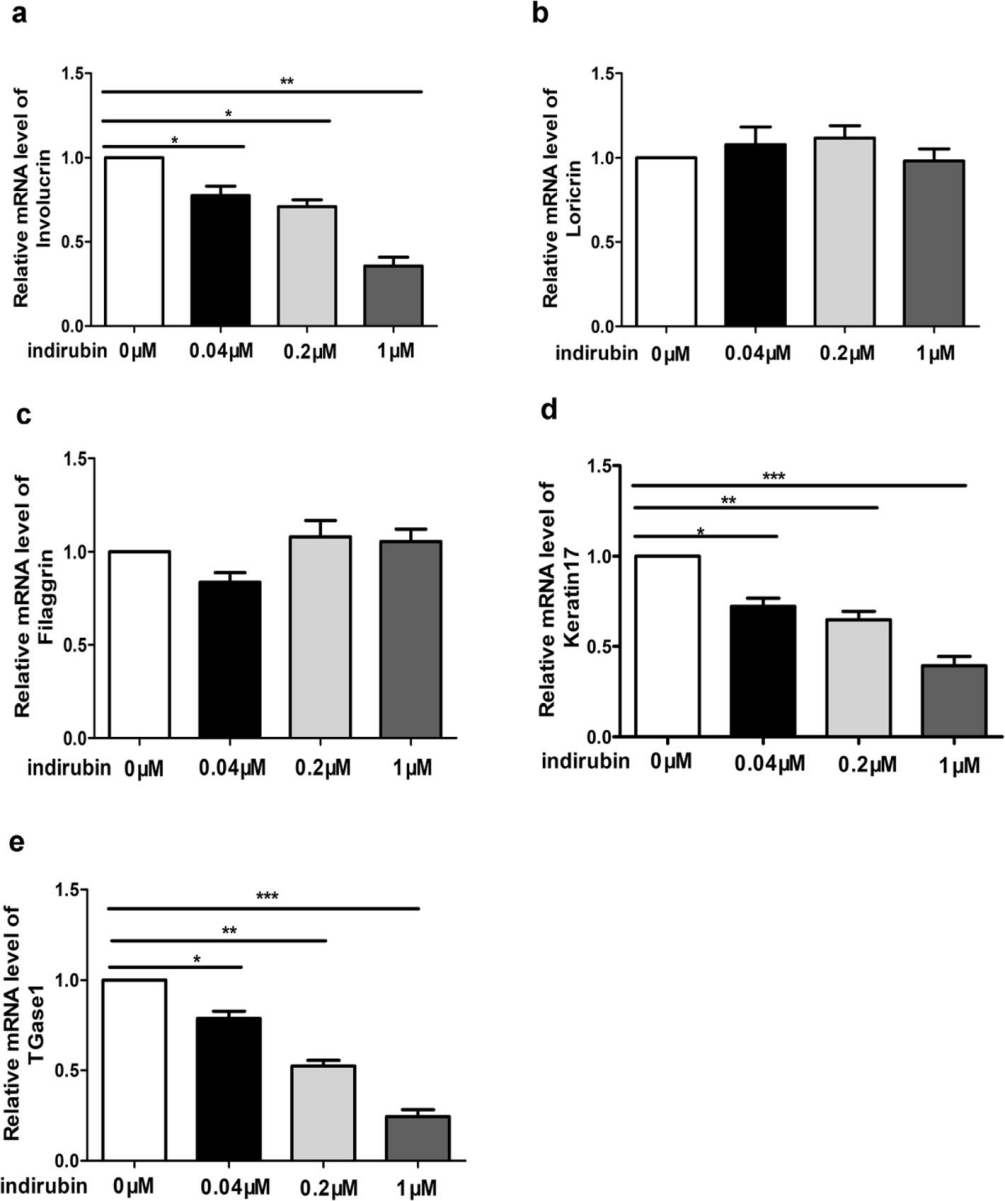
The effects of different concentrations of indirubin on the expression of some proteins in HaCaT cells. The effects of different concentrations (0, 0.04 μ M, 0.2 μ M and 1 μ M) of indirubin on the mRNA expression of Inolucrin(**a**), Loricrin(**b**), Filaggrin(**c**), Keratin 17(**d**) and TGase1(**e**) in HaCaT cells. Indirubin suppresses the mRNA expression of Inolucrin, Keratin 17, TGase1, nevertheless. It barely effects on the mRNA expression of Loricrin and Filaggrin. *P < 0.05, **P < 0.01, ***P < 0.001. (*N* = 3)

We found that indirubin could down-regulate the expression levels of Inolucrin, Keratin 17, and TGase 1 (Fig. 4 a,d, and e). Still, we had no significant effect on the expression of Loricrin and Filaggrin (Fig. 4 b and c) when HaCaT cells were exposed to indirubin (concentrated 0, 0.04 μM, 0.2 μM, and 1 μM) for 48 h and analyzed by qRT-PCR. These results indicate that indirubin can down-regulate the expression of Inolucrin, Keratin 17, and TGase 1 in HaCaT cells, it can improve the differentiation of HaCaT cells.

## Discussion

The wnt signal pathway plays an essential regulatory role in various biological processes such as cell proliferation, survival, migration and polarity, and stem cell self-renewal [17], and it modulates immune/inflammatory cascade. An imbalance of wnt signal pathway function leads to hyper-proliferation and inflammation such as psoriasis [18]. Previous reports indicate that wif-1 is reduced or deleted in a variety of tumor cells, including colon cancer, endometrial cancer and chondrosarcoma. The reduction or deletion expression of wif-1 is due to the wif-1 promoter hypermethylation [19], which promotes the excessive activation of the wnt signal pathway, further promotes cell proliferation and induces apoptosis. The wnt signal pathway is suppressed when the wif-1 expression is restored. Wif-1 is one of the critical inhibitors of wnt signaling. We speculate that wif-1 plays a vital role in the pathogenesis of psoriasis.

In recent years, researchers have found that indirubin has a good effect on topical treatment of psoriasis [7]. It has been confirmed that indirubin can attenuate the epidermal thickness of psoriasis-like skin lesion in mice by regulating CD274 [20]. The improvement of a psoriasis-like skin lesion in mice caused by indirubin has attracted our attention. Considering that indirubin can inhibit cell proliferation, but its potential molecular mechanism in psoriasis has not been fully elucidated. Therefore, we tried to determine whether indirubin could effectively suppress the wif-1 promoter hypermethylation and activate wif-1 expression in HaCaT cells. This effect could modulate the expression of some other well-known wnt signal pathway related protein (Frizzled2, Frizzled5) and β-catenin.

As we hypothesized, we observed that indirubin restored the mRNA and protein expression levels of wif-1 by changing the hypermethylation status of the wif-1 promoter. Further, we uncovered the relationship between DNMT1 and wif-1 promoter methylation. DNMT1 is responsible for the maintenance and propagation of DNA methylation patterns. We discovered DNMT1 promotes wif-1 promoter hypermethylation, the wif-1 promoter is demethylated when DNMT1 is deleted. We also found that indirubin can inhibit the mRNA and protein expression levels of DNMT1. It was proved that DNMT1 could directly inhibit wif-1 activity, and indirubin could enhance wif-1 activity by Luciferase reporter assay. The result showed that indirubin exerted a demethylation effect on wif-1 by inhibiting the expression of DNMT1. These are consistent with the results of Tan M et al., in which inhibition of DNMT expression by miR-29 s restored wif-1 expression in non-small cell lung cancer (NSCLC) [21].

Wnt antagonists (WIF-1and sFRPs) promote the accumulation of nuclear β-catenin and generate a functional transcription factor complex and the expression of downstream target genes when the Wnt signaling pathway is activated. Phosphorylated glycogen synthase kinase-3 (GSK3) facilitates the proteasomal degradation of β-catenin when a wnt signal is absent. β-catenin accumulates in the cytoplasm and is subsequently translocated into the nucleus to switch on the transcription of specific target genes when GSK3 is inhibited by the activation of Dsh. Wnt signaling pathway plays a vital role in chronic inflammatory diseases as psoriasis, such as Il-17A, it inhibits the wnt signal pathway and rescues the expression of wnt target gene and bone formation [22]. A regulation of β-catenin phosphorylation is a vital question in Wnt-signaling transduction. We detected that phosphorylation (Ser33/37/Thr41) level of β-catenin was decreased after exogenously added indirubin in a concentration-dependent manner. The results showed that indirubin inhibited the activity of the wnt signaling pathway by down-regulating the expression level of β-catenin phosphorylation. As mentioned above, when the Wnt signaling pathway is activated, Wnt antagonists (WIF-1 and sFRPs) promote the accumulation of nuclear β-catenin, we have not determined the nuclear transcription level of β-catenin in this study. Still, in the next stage, exploring downstream genes of the wnt signaling pathway, we will examine the problem for β-catenin nucleus translocation.

Besides, we also observed that protein expression and mRNA expression of Frizzled2 and Frizzled5 were inhibited by indirubin. Wif-1 acts as a negative feedback regulator in the wnt/β-catenin pathway, which directly binds to wnt ligands to block wnt/β-catenin signaling, which may be one of the reasons for the decreased expression levels of Frizzled2, Frizzled5 in HaCaT cells. In the research, we showed that indirubin could inhibit the proliferation of HaCaT cells, increase the proportion of cells arrested in the G0/G1 phase, and induce apoptosis of HaCaT cells.

In psoriasis, Involucrin and TGase 1 are proteins expressed in the early stages of keratinocyte differentiation [23, 24]. Involucrin, as a protein precursor of the crosslinked envelope, is a marker of early differentiation of keratinocytes [25]. TGase 1 expression may be regulated by β-catenin and glycogen synthase kinase [26]. Keratin 17 has multiple biological functions and regulates cell proliferation, growth [27], and skin inflammation [28]. In psoriasis, IL-17, IL-22and INF-^3^ stimulate keratin 17 overexpression [29]. Filaggrin and loricrin are proteins expressed in later stages of keratinocyte differentiation [30]. We also found that indirubin can down-regulate the expression levels of Involucrin, TGase 1, and keratin 17 to improve the differentiation of HaCaT cells.

Indirubin inhibits the expression of DNMT1 and restores the expression of wif-1, further, it inhibits wnt / β-catenin signaling, it may be the mechanism by which indirubin inhibits proliferation, arrests cell cycle, induces apoptosis, and differentiation in HaCaT cells. We afforded initial evidence that indirubin reactivates wif-1 from a hypermethylation state and downregulates the wnt/β-catenin canonical pathway.

## Conclusions

Our findings demonstrate that the expression level of wif-1 was recovered after treated with indirubin in HaCaT cells. The expression of wif-1is associated with the hypermethylation status of the wif-1 promoter. The expression of wif-1 was restored by inhibiting the expression of DNMT1 after treatment with indirubin in HaCaT cells. Further, indirubin inhibits the wnt/β-catenin signal pathway. These results suggested that indirubin had inhibited cell proliferation and induced the apoptosis of HaCaT cells, reflecting its potential use in the treatment of proliferative diseases such as psoriasis.

## Data Availability

The datasets used and analyzed in the current study are available from the corresponding author upon reasonable request.

## Abbreviations

DNMT1: DNA methyltransferase 1
wif-1: wnt inhibitory factor 1
ELISA: enzyme-linked immunosorbent assay
CCK8: Cell Counting Kit-8
TGase1: transcriptional activation of transglutaminase 1
EGFR: Epidermal growth factor receptor
qRT-PCR: quantitative real-time polymerase chain reaction
DMEM: Dulbecco’s Modified Eagle's medium
FBS: fetal bovine serum
DMSO: dimethyl sulfoxide
GAPDH: glyceraldehyde-3-phosphate dehydrogenase
MSP: methylation-specific PCR
FITC: Annexin V-fluorescein isothiocyanate
PI: propidium iodide
PBS: phosphate-buffered saline
NSCLC: non-small cell lung cancer
SPSS: Statistical Product and Service Solutions
sFRPs: secreted frizzled-related proteins
LDL: low-density lipoprotein
LRP5/LRP6: receptor-related protein 5 and 6
GSK3: glycogen synthase kinase-3
